# The Antihelminthic Niclosamide Inhibits Cancer Stemness, Extracellular Matrix Remodeling, and Metastasis through Dysregulation of the Nuclear β-catenin/c-Myc axis in OSCC

**DOI:** 10.1038/s41598-018-30692-3

**Published:** 2018-08-24

**Authors:** Lin-Hong Wang, Mei Xu, Luo-Qin Fu, Xiao-Yi Chen, Fan Yang

**Affiliations:** 1 Department of Stomatology, Zhejiang Provincial People’s Hospital, People’s Hospital of Hangzhou Medical College, Hangzhou, 310014 Zhejiang Province China; 2 Special Department, Hangzhou Dental Hospital, Hangzhou, 310013 Zhejiang Province China; 3 Clinical Research Institute, Zhejiang Provincial People’s Hospital, People’s Hospital of Hangzhou Medical College, Hangzhou, 310014 Zhejiang Province China; 4 Key Laboratory of Tumor Molecular Diagnosis and Individualized Medicine of Zhejiang Province, Hangzhou, 310014 Zhejiang Province China

## Abstract

Niclosamide is an oral chlorinated salicylanilide antihelminthic agent with potential anticancer activity suggested in several cancer types, however, its anticancer action and likely molecular mechanism in malignant oral cells remain unclear. In the present study, we demonstrated that ALDH+ human oral squamous cell carcinoma (OSCC) cells are characterized by upregulated expression of the pluripotency transcription factors OCT4, Nanog and Sox2, as well as exhibit enhanced cancer stemness, as demonstrated by enhanced tumorsphere formation. We also showed that niclosamide effectively inhibits activation of the Wnt/β-catenin signaling pathway by targeting multiple components of this pathway, including downregulating the expression β-catenin, Dishevelled 2 (DVL2), phosphorylated glycogen synthase kinase-3β (p-GSK3β) and Cyclin D1, in human OSCC SCC4 and SCC25 cell lines, as well as reduced the formation of primary and secondary tumorspheres. In addition, we showed that niclosamide inhibits the epithelial-to-mesenchymal transition (EMT), migration and colony formation of the OSCC cells, by dose-dependently upregulating E-cadherin and the tissue inhibitor of metalloproteinases 2 (TIMP2) mRNA levels, while reducing the expression levels of vimentin, snail, MMP2 and MMP9 mRNA. These anticancer activities of niclosamide were similar to those caused by interference with nuclear β-catenin/c-Myc expression using the siRNA transfection. Finally, we demonstrated that niclosamide inhibits cisplatin-induced OSCC stem cell enrichment and enhances sensitivity to cisplatin in ALDH+ tumorspheres. These experimental data, combined with accumulated evidence, are suggestive of the potential and efficacy of niclosamide in the treatment of OSCC.

## Introduction

Oral cancer, consisting mostly of oral squamous cell carcinoma (OSCC), is one of the most common malignancies globally. Unlike the other subtypes of oral cancer, OSCC is characterized by high incidence, relatively low 5-year survival rate, low sensitivity to chemotherapy, development of resistance to chemo- or radio-therapy and subsequent treatment failure^1–3^. The current therapy of choice, which is the docetaxel (DTX), cisplatin (CDDP), and 5-flurouracil (5-FU) combination chemotherapy, especially for advance-stage malignancies, is often beleaguered with enhanced risk of severe drug-related adverse effects, organ toxicity, and little survival benefits^4^.

The presence and activities of cancer stem stem cells (CSCs) or so-called tumor initiating cells (TICs) among the oral cancer cells has been implicated in its low response to chemotherapy and poor prognosis^5,6^. These oral CSCs are perpetually self-renewing, highly proliferative, tumorigenic even at low-cell density, drive tumor aggression, resistance to standard chemotherapy and radiotherapy^6^. Like the embryonic stem cells (ESCs), oral CSCs are characterized by their expression of pluripotency transcription factors such as Oct4, Nanog, Sox2, extracellular expression of CD44+/CD24−, and elevated enzymatic activity of aldehyde dehydrogenase (ALDH)^6,7^. All the aforementioned, added to the high likelihood of distant metastases and local recurrence^3^, necessitate the discovery or development of novel and highly efficacious anticancer agents which effectively targets these oral CSCs, and/or regulate the CSC-associated signaling pathways.

The aberrant activation of the Wnt/β-catenin signaling is a common theme across several cancer types, including OSCC; more importantly, aberrantly increased Wnt/β-catenin expression or activity has been implicated in CSCs biology^8,9^, making it a potential molecular target and promising approach for regulation of the CSCs and prevention of oral CSC - facilitated metastasis and recurrence.

Niclosamide is an old drug with potential new application, as it effectively enhances the degradation of LRP6^10^, induce the internalization of Frizzled 1 (Fz)^11^, and downregulate Wnt signaling, and has been shown to elicit antitumor responses in several tumors^10,11^. Niclosamide is an inexpensive and safe FDA-approved oral chlorinated salicylanilide antihelminthic/teniacidal agent with potential anticancer activity suggested in several cancer types, including acute myelogenous leukemia^12^, colon^13^, and ovarian^14^ cancers by high-throughput screening. Consistently, the effect of niclosamide on CSCs has been observed in other types of cancer such as leukemias and breast cancers^15,16^. More recently, it was reported that a niclosamide loaded rigid core mixed micelle could selectively reduce the CD44+ CSCs population in cutaneous melanoma cells^17^.

In this present study, we demonstrated that ALDH+ human OSCC cells are characterized by upregulated expression of the pluripotency transcription factors OCT4, Nanog and Sox2, as well as exhibit enhanced cancer stemness, as demonstrated by enhanced tumorsphere formation, *in vitro*. Importantly, we also showed that niclosamide effectively inhibits the activation of the Wnt/ β-catenin signaling pathway by targeting multiple components of this pathway, including downregulating the expression β-catenin, DVL2, p-GSK3β and cyclin D1, in human OSCC SCC4 and SCC25 cell lines, as well as reduced the formation of primary and secondary tumorspheres. These molecular events were correlated with the niclosamide-induced suppression of MMP2, MMP9, Vimentin and Snail expression, as well as the converse upregulation of E-cadherin and the tissue inhibitor of metalloproteinases 2 (TIMP2) expression levels in ALDH+ cells. Additionally, we demonstrated that niclosamide inhibits cisplatin-induced oral cancer stem cell enrichment and enhances sensitivity of the ALDH+ OSCC cells to cisplatin treatment.

## Materials and Methods

### Drugs and reagents

Niclosamide (N3510 SIGMA, ≥98% TLC, Sigma-Aldrich, St. Louis, MO, USA) was suspended in DMSO, prepared at a stock concentration of 1 mg/ml and stored at 4 °C. Cisplatin (479306 ALDRICH, ≥99.9% trace metal basis, Sigma-Aldrich, St. Louis, MO, USA) was dissolved in PBS, prepared at a stock solution of 1 mM and stored at −20 °C away from light. The ALDEFLUOR kit was obtained from StemCell Technologies (Vancouver, Canada). Antibodies against Oct4 (sc-5279), Nanog (sc-376915), Sox2 (sc-365964), Snail (sc-28199), c-Myc (sc-40), Notch1 (sc-71719), β-catenin (sc-65480), MMP-2 (sc-13594), MMP-9 (sc-21733) and TIMP-2 (sc-21735) were purchased from Santa Cruz Biotechnology, Inc, while anti-Dishevelled 2 (ab22616), GSK3β (phospho Y216, ab75745), Cyclin D1 (ab16663), GAPDH (ab8245) and β-actin (ab6276) were obtained from Abcam.

### Cell lines and cell culture

The human oral squamous cell carcinoma cell lines, SCC4 and SCC25 were originally obtained from the American Type Culture Collection (ATCC, Rockville, MD) and were cultured in Dulbecco’s modified Eagle’s medium/F12 (DMEM/F12, Gibco, Carlsbad, CA) supplemented with 10% fetal bovine serum (FBS), 2 mM L-glutamine 100 U/ml penicillin and 100 g/ml streptomycin (Thermo Fisher Scientific, Inc. Waltham, MA) in a 5% CO_2_ humidified atmosphere at 37 °C. Cultured cells were passaged at 98% confluence or medium changed every 72 h.

### Tumor sphere formation assay

SSC4 or SSC25 cells were exposed to 0 μM (control) or 10 μM (treated) of niclosamide for 48 h. Tumorspheres were then generated by plating 1 × 10^4^ niclosamide-treated or untreated control SCC4 or SCC25 cells per well in ultra-low adhesion 6-well plates, containing 2 mL warm StemXVivo serum-free tumorsphere media (R&D Systems, Minneapolis, MN) supplemented with 2 U/mL heparin (Sigma) and 0.5 g/mL hydrocortisone (Sigma) following the manufacturer’s protocol, then incubated in 5% CO_2_ incubator, at 37 °C for 12 days. Tumorspheres were observed under microscope, those larger than 50 microns counted, and image taken.

### TOPflash Luciferase Reporter Assay

To assess the transcriptional activity of β-catenin in oral squamous cell carcinoma cells, we employed the TOP/FOP reporter system using the dual-luciferase kit (Dual-GloTM Luciferase Assay System, Promega, Madison, WI). TOPFlash contains three copies of the TCF/LEF binding site [AAGATCAAAGGGGGT] upstream of the thymidine kinase minimal promoter, and FOPFlash contains a mutated TCF/LEF binding site [AAGGCCAAAGGGGGT]. The underlined nucleotides were mutated in FOPFlash-luciferase reporter construct. Sorted ALDH+ SCC4 or SCC25 cells were plated at 4 × 10^4^ cells/well and incubated in 5% CO_2_ incubator at 37 °C for 24 h. The cells were then transfected with 250 ng of TCF/LEF activity reporter (TOPFlash and FOPFlash), using Lipofectamine™2000 (Invitrogen, Carlsbad, CA) in Opti-MEM (Gibco) according to the manufacturer’s instructions. The results are expressed as a ratio of TOPFlash:FOPFlash normalized to β-galactosidase (50 ng) which served as an internal control for transfection efficiency. Six hours after plasmid transfection, the ALDH+ OSCC cells were treated with 2.5–10 μM of niclosamide for 24 h. SCC4− or SCC25− derived tumorspheres were plated and transfected in the similarly. Cells were harvested, and total protein obtained 24 hours after treatment and the luciferase activity assessed by using a Synergy 2 plate reader (Biotek Instruments Inc., VT). The luciferase reading was normalized to the firefly luciferase activity for all the wells. Relative luciferase activity (in arbitrary units) was reported as fold induction after normalization for transfection efficiency. The normalized luciferase activity from experiments performed in triplicate was compared between the experimental and control groups for statistical significance with unpaired Student’s t test.

### ALDEFLUOR assay

OSCC cells with enhanced ALDH activity were identified, evaluated and isolated using the ALDEFLOUR^TM^ assay kit (StemCell Technologies, Vancouver, Canada) according to the manufacturer’s instruction. In brief, 1 × 10^6^ OSCC cells were incubated in Aldefluor® assay buffer containing a 1.5 μM ALDH substrate for 30 min at 37 °C. Each sample was treated with 50 μM of DEAB and used as a negative control. Prior to analysis, cells were stained with 1 mg/ml of propidium iodide to evaluate their viability. The fluorescence intensity of the stained cells was analysed using a FACSAria cell sorter Flow Cytometer (BD Biosciences). The reaction with DEAB was used to define the baseline for the assay. The ALDH activity of a sample was determined to be ‘high’ or ‘low’ based on the fluorescence intensity beyond or below the threshold defined by the reaction with DEAB. The purity of FACS-sorted ALDH1 ‘high’ or ‘low’ cells as the 98% and 99%, respectively. 7-amino-actinomycin D (7-AAD; BD Biosciences) was added at a final concentration of 0.25 μg/ml for exclusion of nonviable cells.

### Transfection with β-catenin siRNA

2.5 × 10^5^ ALDH+ SCC4 cells were plated per well in 6-well plates in triplicates and cultured to approximately 50% confluence. The cells were then incubated in 2 mL serum-free DMEM/F12 with Lipofectamine 2000 containing the β-catenin siRNA, for siRNA transfection. Six hours later, the medium was replaced with complete DMEM/F12 culture medium supplemented with 10% FBS and incubated for 72 hours. Analysis of gene expression based on isolated mRNA or protein was then performed. Cells incubated with Lipofectamine 2000 without any siRNA were used as control. The negative control siRNA and siRNA targeting the β-catenin gene used in the present study was purchased from Shanghai GenePharma Co., Ltd. (Shanghai, China).

### Western blot analysis

10% polyacrylamide sodium dodecyl sulfate - polyacrylamide gel electrophoresis (SDS-PAGE) was performed using 20 μg of total cell lysates and the blots were transferred onto polyvinylidene difluoride (PVDF) membranes. The membranes with the blots were then incubated with 5% non-fat milk in PBS with Tween-20 (PBST) for 1 h followed by overnight incubation at 4 °C in specific primary antibodies against β-catenin, DVL2, p-GSK3β, Cyclin D1, Snail, Vimentin, c-Myc, Sox2, Oct4, Nanog and β-actin, then incubated in peroxidase - conjugated secondary antibody for 1 h at room temperature, washed with PBST three times, and the protein signals were analyzed with the enhanced chemiluminescent reagents (Millipore).

### Colony formation assay

After cell sorting, ALDH+ or ALDH− SCC4 or SCC25 cells pre-treated with niclosamide or/and cisplatin for 48 h were plated in triplicate at 200 cells/well in the 6-well plates and cultured for 12–15 days. When grown colonies consisted of >50 cells, they were carefully washed with PBS twice, fixed with methanol for 15 min, and stained with crystal-violet for 15 min at room temperature, then observed and counted under microscope. The number of cells within a colony was manually counted in the case of small colonies of up to a number of 50 cells. In the case of bigger colonies, the number was estimated by counting the number of cells within a representative encircled part of the colony and subsequently extrapolated to the surface of the colony. The difference in colony number and size of treated cells were correlated with those of the untreated control group.

### *In vitro* invasion and migration assays

For invasion, the BioCoat Matrigel Invasion Chamber was used, and procedure was performed following the manufacturer’s protocol. Niclosamide-treated or untreated control, as well as β-catenin siRNA - transfected or sham/control siRNA transfected OSCC cells were plated on the Matrigel coat of the upper chamber culture inserts at 1.5 × 10^5^ cells/500 *μ*l of serum-free DMEM/F12, while the lower chamber contained 10% FBS - supplemented complete DMEM culture medium and incubated in a 5% CO_2_ incubator at 37 °C for 24 h. Then the non-invaded cells on the upper surface of the membrane were carefully removed using cotton swabs, and the invaded cells on the lower surface of the membrane were fixed with methanol and stained with crystal violet. Five randomly selected microscopic fields (×200) per membrane was selected and the numbers of stained cells were counted. For the transwell migration assay, 3 × 10^4^ cells were plated in the upper chamber harboring non-coated membrane (24-well insert; 8-μm pore size; BD Biosciences). After incubation for 24 h, migrated cells were stained, image taken and quantified at an OD of 560 nm.

### Quantitative real-time PCR

The Oct4, Nanog, Sox2, Notch1, E-cadherin, Vimentin, and Snail mRNA expression levels in the sorted ALDH+ or ALDH− OSCC cells, as well as in the niclosamide-treated or untreated control cells were estimated using RT-qPCR. After the extraction of total cellular RNA using TRIzol reagent (Invitrogen, Carlsbad, CA), we reverse-transcribed 1 μg of the total RNA using the QuantiTect Reverse Transcription Kit (Qiagen, Germantown, MD). Quantification of mRNA expression was performed with the LightCycler 480 System (Roche LifeScience, Mannheim, Germany), and the amplified mRNA level of each specific mRNA was normalized to GAPDH. All procedure was performed following manufacturers’ instruction. The following primers were used: Oct4 (forward: 5′-ACATCAAAGCTCTGCAGAAAGAACT-3′; reverse: 5′-CTGAATACCTTCCCAAATAGAACCC-3′); Nanog (forward: 5′-ACATGCAACCTGAAGACGTGTG-3′; reverse: 5′-CATGGAAACCAGAACACGTGG-3′); Sox2 (forward: 5′-CAGGAGAACCCCAAGATGCACAA-3′; reverse: 5′-AATCCGGGTGCTCCTTCATGTG-3′); Notch1 (forward: 5′-GCAGTTGTGCTCCTGAAGAA-3′; reverse: 5′-CGGGCGGCCAGAAAC-3′); b-actin (forward: 5′-CATGTACGTTGCTATCCAGGC-3′; reverse: 5′-CTCCTTAATGTCACGCACGAT-3′); MMP-2 (forward: 5′-CCAAGGTCAATGTCAGGAGAG-3′; reverse: 5′-GCACCCATTTACACCTACAC-3′); MMP-9 (forward: 5′-TGTACCGCTATGGTTACACTCG-3′; reverse: 5′-GGCAGGGACAGTTGCTTCT-3′); TIMP1 (forward: 5′-TTCCGACCTCGTCATCAGGG-3′; reverse: 5′-TAGACGAACCGGATGTCAGC-3′); E-cadherin (forward: 5′-TGGAGGAATTCTTGCTTTGC-3′; reverse: 5′-CGTACATGTCAGCCAGCTTC-3′); Vimentin (forward: 5′-TCTCTGAGGCTGCCAACCG-3′; reverse: 5′-CGAAGGTGACGAGCCATTTCC-3′); Snail (forward: 5′-CCCCAATCGGAAGCCTAACT-3′; reverse: 5′-GCTGGAAGGTAAACTCTGGATTAGA-3′) and GAPDH (forward: 5′-AATCCCATCACCATCTTCCA-3′; reverse: 5′-TGGACTCCACGACGTACTCA-3′). The cycling conditions were as follows: initial denaturation at 98 °C for 5 min, followed by 45 cycles at 98 °C for 15 s, 60 °C for 30 s and 72 °C for 60 s. The experiments were performed in triplicate.

### Statistical analyses

SPSS v.18.0 for Windows software (SPSS Inc. Chicago, IL) was used for statistical analysis. All data are expressed as mean + SEM of experiments performed independently at least twice in triplicate. One-way ANOVA and student’s *t*-test were used to determine the statistical differences between treatment groups. A p value < 0.05 was considered statistically significant.

## Results

### ALDH+ human oral squamous cell carcinoma cells are characterized by upregulated expression of pluripotency transcription factors and exhibit enhanced cancer stemness

**Figure 1:**
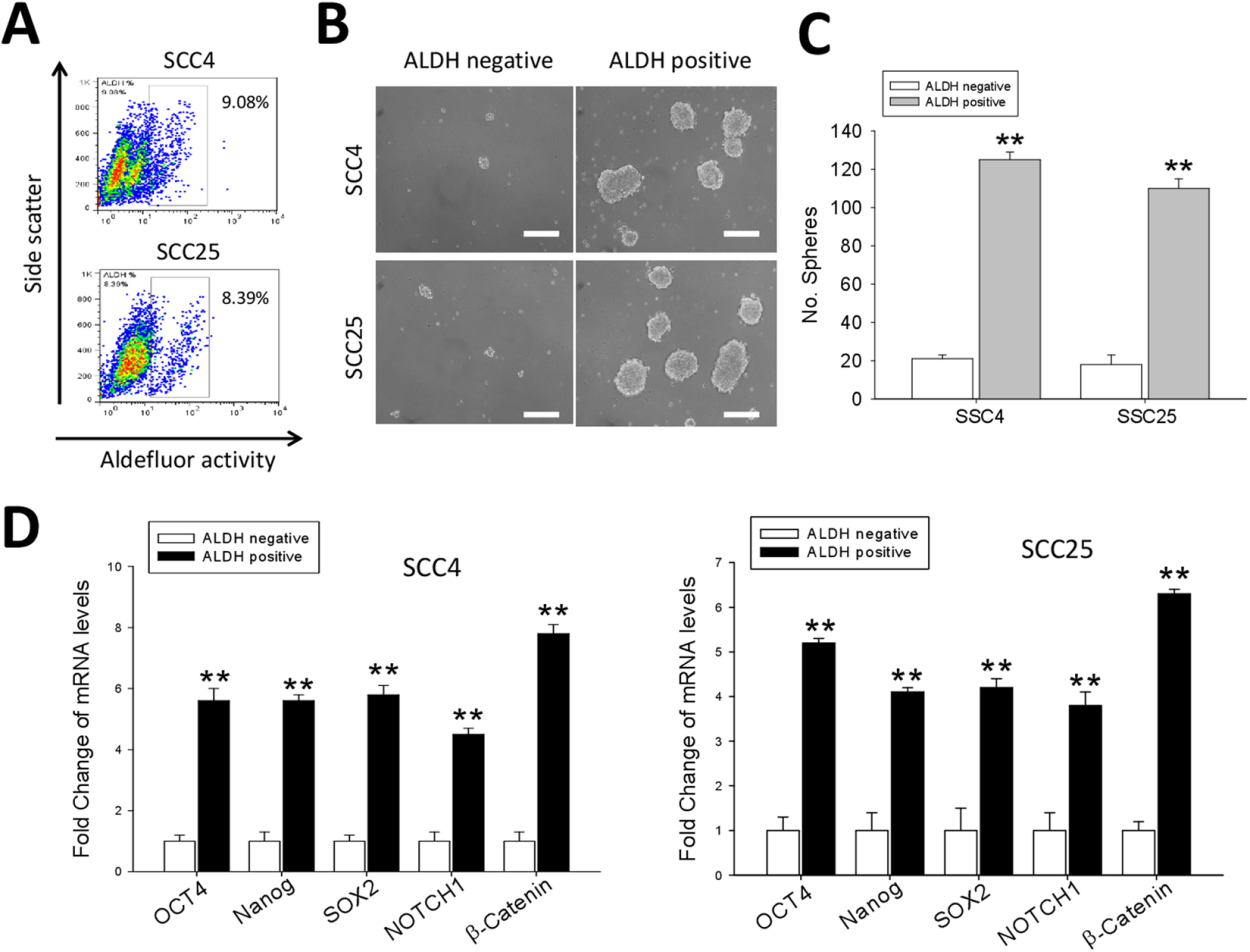
ALDH+ human oral squamous cell carcinoma cells are characterized by upregulated expression of pluripotency transcription factors and exhibit enhanced cancer stemness. (**A**) ALDEFLUOR-based sorting of SCC4 and SCC25 oral cancer cells into ALDH+ and ALDH− cell populations. (**B**) Representative tumorsphere formation assay images showing significantly enhanced tumorsphere-forming potential in the ALDH+ cells compared to the ALDH− group. Scale bar = 50 μm. (**C**) Graph showing that the ALDH+ cells formed significantly more tumorspheres than their ALDH− counterparts, p < 0.01 for both SCC4 and SCC25 ALDH+ *vs* ALDH− cells. (**D**) Graphical representation of the Oct4, Nanog, Sox2, Notch1 and β-catenin mRNA profile in ALDH+ SCC4 or SCC25 cells compared to their ALDH− counterparts. Data represents experiments performed in triplicates and expressed as mean ± SD. *p < 0.05, **p < 0.01, ***p < 0.001.

Since there is increasing evidence that ALDH is a marker for CSCs in both solid and hematologic malignancies, we first characterized the human oral squamous cell carcinoma cells, SCC4 and SCC25, used in our study on the basis of their ALDH expression, selected mRNA expression profile and associated phenotype. Our data indicate that 9.08% and 8.39% of the SCC4 and SCC25 cells, respectively, were ALDH+ based on their Aldefluor activity (Fig. 1A). After isolation of the ALDH+ and ALDH− cells, we performed the tumorsphere formation assay to evaluate and compare the tumorsphere-forming potential of the ALDH+ and ALDH− OSCC cells, and by inference, the self-renewal capacity. Our data showed that the number of tumorspheres formed by the ADLH+ cells were significantly more in number and much larger in size than those by the ALDH− cells (both cell lines: p < 0.01) (Fig. 1B and C). The results of our assessment of the mRNA levels of genes involved in cancer stemness and pluripotency in the OSCC cells demonstrated that compared with their expression in the ALDH− SCC4 or SCC25 cells, there was significantly increased expression levels of Oct4 (p < 0.01), Nanog (p < 0.01), Sox2 (p < 0.01), Notch (p < 0.01) and β-catenin (p < 0.01) in the ALDH+ cells (Fig. 1D). Interestingly, we investigated WNT/β-catenin expression in OSCC using the open access cancer microarray database Oncomine (https://www.oncomine.org/resource/login.html). Box-plot diagrams were analyzed to compare the WNT/β-catenin mRNA levels in normal tongue mucosa tissue with that in tongue squamous cell carcinoma using the Oncomine dataset. The results revealed that WNT/β-catenin expression at the mRNA level was 1.377-fold higher in tongue squamous cell carcinoma (TSCC) than that in normal tongue mucosa tissues (P = 0.002). Kaplan–Meier curves for TGCA database, suggested worse overall survival in patients with high versus low WNT/CTNNB1 expression (Supplementary Fig. S1). These results suggest that WNT/β-catenin upregulation may play a crucial role in oral squamous cell carcinoma development.

### The cancer stem cell-like trait of ALDH-rich oral cancer cell lines is significantly suppressed by niclosamide

**Figure 2:**
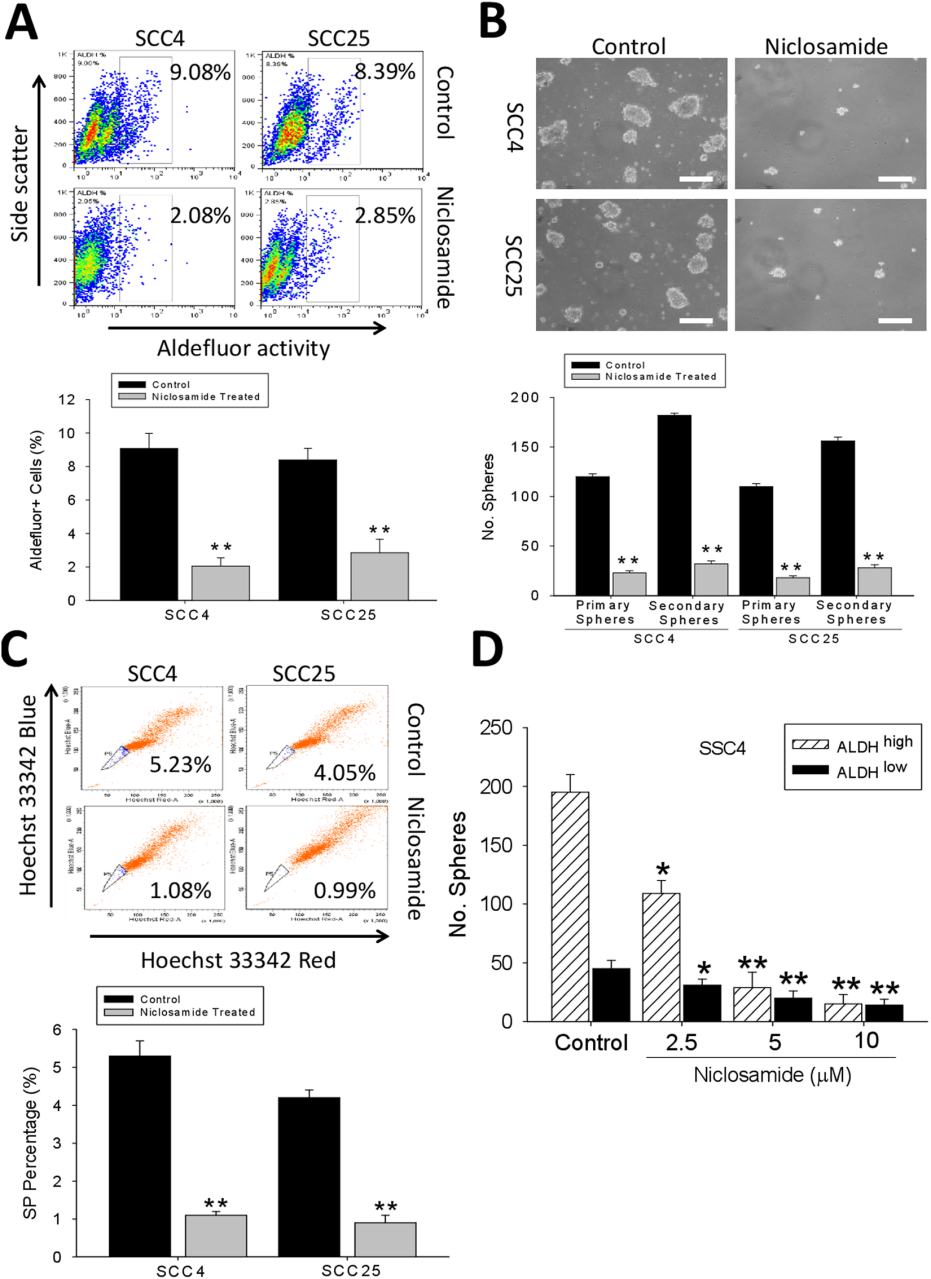
The cancer stem cell-like trait of ALDH-rich oral cancer cell lines is significantly suppressed by niclosamide. (**A**) Representative data showing the effect of pre-treating SCC4 and SCC25 cells with 0 μM (control) or 10 μM (treated) niclosamide for 48 h, on their ALDH activity as detected by flow cytometry-based ALDEFLUOR assay (*upper*), and the graphical quantitative analysis of treated ALDH+, compared to untreated control cells (*lower*). (**B**) The inhibitory effect of niclosamide pre-treatment on the formation of OSCC tumorspheres from SCC4 and SCC25 (*upper*), and quantitative representation of the effect of niclosamide treatment on the formation of primary and secondary tumorspheres from SCC4 and SCC25 cells (*lower*), scale bar = 20 μm. (**C**) OSCC side population is reduced in the niclosamide-treated SCC4 and SCC25 cells, compared to the untreated control cell group. (**D**) Number of secondary spheres in ALDHhigh and ALDHlow SSC4 cells treated with Niclosamide. Data represents experiments performed in triplicates and expressed as mean ± SD. *p < 0.05, **p < 0.01, ***p < 0.001.

Based on its suggested anticancer activity in acute myelogenous leukemia^12^, colon^13^, and ovarian^14^ cancers, we examined whether niclosamide exhibits similar cytotoxic effect against the adherence-independent ALDH+ OSCC cells. We observed that treatment with 10 μM of niclosamide caused 4.4 - and 2.9 -fold reduction in the Aldefluor activity of the ALDH+ SCC4 (p < 0.01) and SCC25 (p < 0.01) cells, respectively (Fig. 2A). Also, we demonstrated that the same concentration of niclosamide induced a significant suppression of the ALDH+ OSCC cells to form tumorspheres, quantity- and size-wise (Fig. 2B, *upper*). Additionally, we noted that this inhibitory effect of niclosamide was not restricted to the primary tumorspheres alone, but also in the secondary generation; as we recorded a 83.3% (p < 0.01) and 85.8% (p < 0.01) decrease in the number of primary and secondary tumorspheres formed, respectively, in the niclosamide-treated SCC4 cells, compared with the untreated control cells, while for the niclosamide-treated SCC25 cells, the number of primary and secondary tumorspheres formed were reduced by 83.6% (p < 0.01) and 83.3% (p < 0.01), respectively, compared with the untreated control cells (Fig. 2B, *lower*). Consistent with this data, we further demonstrated that treatment with 10 μM of niclosamide significantly reduced the quantity of Hoechst 33342 red/Hoechst 33342 blue - stained SCC4 and SCC25 side population cells by 4.8 - (p < 0.01) and 4.1 - (p < 0.01) fold, respectively, compared with the control group (Fig. 2C). Moreover, niclosamide drastically dose-dependent reduced the self-renewal ability of ALDH^high^, and only marginally that of ALDH^low^ spheres (Fig. 2D), consistent with lower SOX2 levels in the ALDH^low^ subpopulation. These findings are indicative of the tumor-killing and self-renewal limiting effects of niclosamide in OSCC cell lines and confirm the critical role of targeting oral CSCs in niclosamide anticancer activity.

### Niclosamide inhibits the migration and epithelial-to-mesenchymal transition of oral squamous cell carcinoma cells

**Figure 3:**
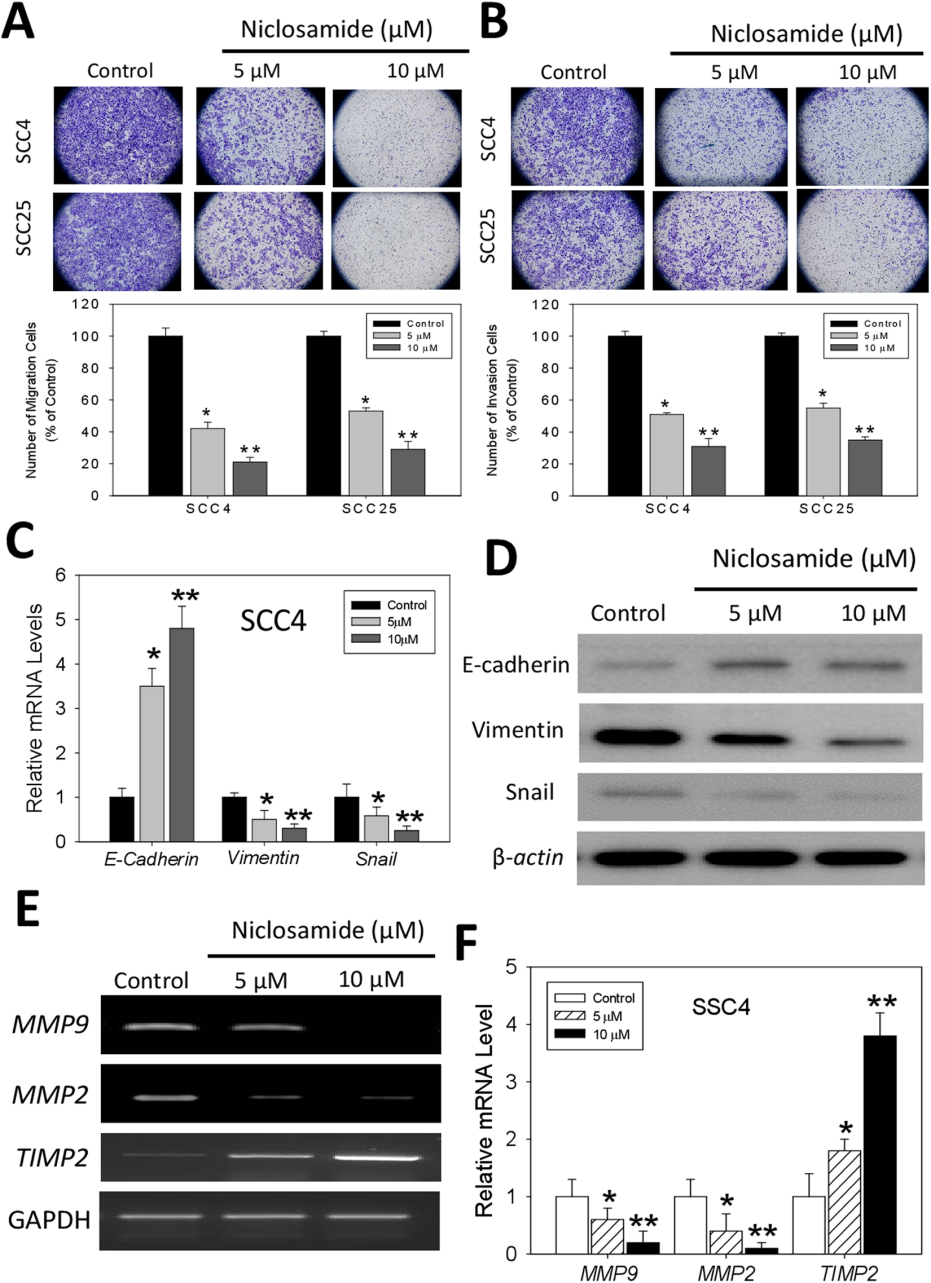
Niclosamide inhibits the migration and epithelial - to - mesenchymal transition of oral squamous cell carcinoma cells. (**A**) Representative image showing that treatment with niclosamide significantly inhibits the migration of SCC4 and SCC25 OSCC cells in a dose-dependent manner (*upper*). Graphical quantitative data of the relative number of migrated cells in niclosamide-treated cells, compared to untreated control cells (*lower*). (**B**) Niclosamide suppresses the invasive potential of OSCC cells (*upper*). Graphical quantitative data showing the relative number of invaded cells in niclosamide-treated cells, compared to untreated control cells (*lower*). (**C**) Effect of niclosamide treatment on the expression of E-cadherin, Vimentin and Snail mRNA expression levels. (**D**) Western blot analyses showed that the protein expression of Vimentin and Snail was decreased with a dose-dependent manner in SCC4 cells treated with niclosamide. In contrast, the expression of E-cadherin was significantly increased. (**E**) Treatment with niclosamide downregulates MMP9 and MMP2 but upregulates TIMP2 mRNA expression level. GAPDH was used as internal control. (**F**) The bar graphs represent the densitometric measurements of the bands seen by RT-PCR procedures. Data represents experiments performed in triplicates and expressed as mean ± SD. *p < 0.05, **p < 0.01, ***p < 0.001.

To better understand the anticancer activities of niclosamide, we investigated its effect on the characteristic aggressive phenotype of the ALDH+ OSCC cells. We demonstrated that 48 h treatment of the SCC4 and SCC25 cells with 5–10 μM of niclosamide significantly inhibited the ability of the cancer cells to migrate in a dose-dependent manner. While a 60% (p < 0.05) and 81% (p < 0.01) decrease in number of migrated cells was observed in the SCC4 cells treated with 5 or 10 μM of niclosamide, compared with the untreated control cells; for the 5 or 10 μM niclosamide - treated SCC25 cells, a 54% (p < 0.05) and 70% (p < 0.01) reduction was noted, respectively, compared with the control group (Fig. 3A). Furthermore, using the matrigel invasion assay, we demonstrated that treatment with 5 or 10 μM of niclosamide induced a significant dose-dependent reduction in the ability of the SCC4 and SCC25 cells to invade through the matrigel, as expressed by a 50% (p < 0.05) and 68% (p < 0.01) in SCC4 cells treated with 5 or 10 μM of niclosamide, respectively, compared with the untreated control cells, while for the SCC25 cells, 48% (p < 0.05) and 66% (p < 0.01) reduction in invasion was noted (Fig. 3B). In addition, our results indicate that the expression levels of vimentin and snail mRNAs were markedly inhibited in SCC4 cells after niclosamide treatment, while conversely, E-cadherin mRNA expression level was enhanced (Fig. 3C). Western blot analyses showed that a dose-dependent up-regulated expression of E-cadherin in SCC4 cells following treatment with niclosamide, while the expression of Vimentin protein was significantly down-regulated. In addition, a decreased expression of Snail in a dose-dependent manner were observed (Fig. 3D). Similarly, treatment with 5–10 μM of niclosamide upregulated TIMP2 mRNA expression levels, while downregulating the expression of MMP9 and MMP2 mRNA levels, in a dose-dependent manner (Fig. 3E and F). These data indicate a potential inhibitory role for niclosamide against oncogenic ECM remodeling, EMT, migration and invasion of OSCC cells.

### The anticancer activity of nuclosamide is mediated by the dysregulation of the Wnt/β-Catenin signaling pathway

**Figure 4:**
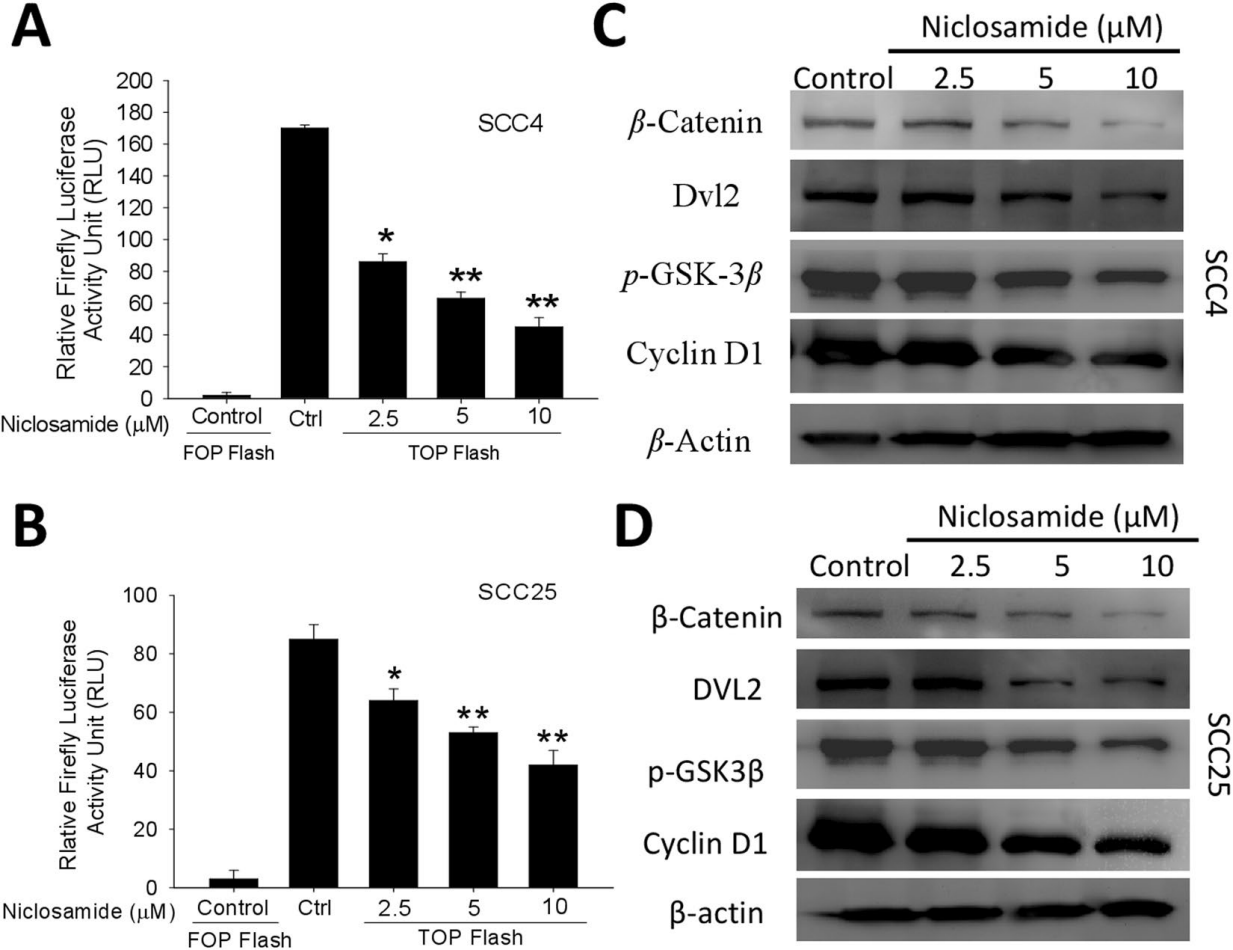
The anticancer activity of nuclosamide is mediated by the dysregulation of the Wnt/β-Catenin signaling pathway. TCF/LEF plasmid reporter activity was evaluated in (**A**) SCC4 and (**B**) SCC25 cells treated with different concentrations of niclosamide, using the TOPflash assay. Representative western blot data showing the inhibitory effect of niclosamide on the expression levels of β-catenin, DVL2, p-GSK3β, and Cyclin D1 proteins in (**C**) SCC4 and (**D**) SCC25 OSCC cells. β-actin was used as loading control. Data represents experiments performed in triplicates and expressed as mean ± SD. *p < 0.05, **p < 0.01, ***p < 0.001.

Using the TOPFlash reporter system, we identified niclosamide as an active inhibitor of the Wnt/β-catenin signaling pathway. Niclosamide effectively inhibited the TOPflash reporter gene in a dose-dependent manner but had no effect on the FOPflash (harboring mutant TCF binding site) activity in the ALDH+ SCC4 (Fig. 4A) and SCC25 (Fig. 4B) OSCC cells, thus, suggesting that the observed niclosamide inhibitory activity was Wnt/β-catenin/TCF signaling axis - dependent. Consistent with this observed Wnt inhibitory effect of niclosamide, our western blot assay results further demonstrated that treatment with 2.5–10 μM of niclosamide downregulated the expression levels of β-catenin, DVL2, p-GSK3β and Cyclin D1 proteins in the treated ALDH+ SCC4 (Fig. 4C) and SCC25 (Fig. 4D) OSCC cells, compared with the untreated cells.

### Downregulated β-catenin expression is critical for inhibition of the cancer stemness, migration, invasion and colony-formation of ALDH+ oral cancer cells

**Figure 5:**
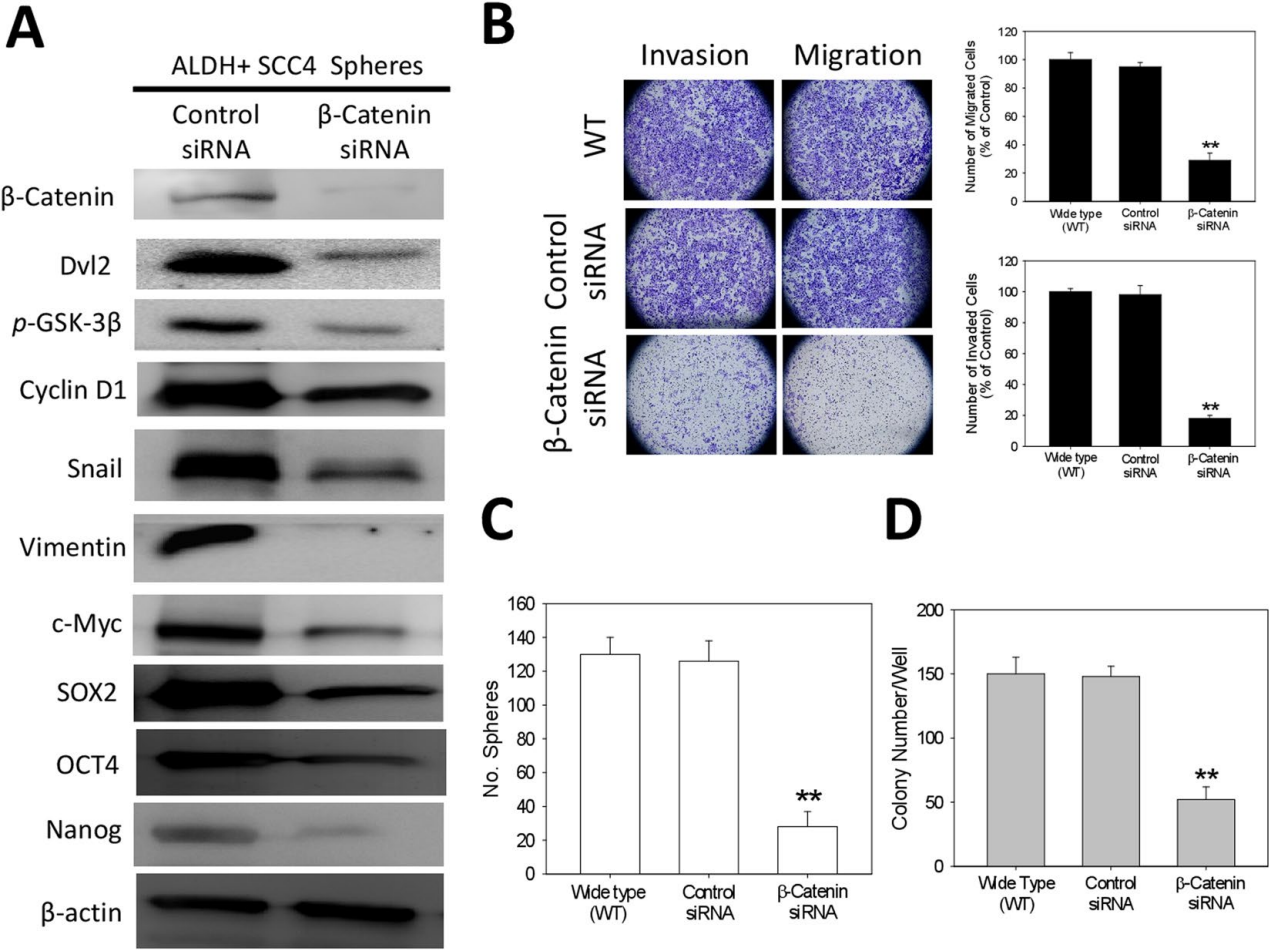
Downregulated β-Catenin expression is critical for inhibition of the cancer stemness, migration, invasion and colony-formation of ALDH+ oral cancer cells. (**A**) Reduced expression levels of β-catenin, Snail, c-Myc, Cyclin D1, Sox2, Oct4 and Nanog proteins in tumorspheres derived from ALDH+ SCC4 cells transfected with β-catenin-specific siRNA, compared with control siRNA-transfected group, using western blot assays. (**B**) Images showing that the number of invaded and migrated ALDH+ SCC4 cells was significantly decreased in the β-catenin-specific siRNA group, compared to the control siRNA or wild type cell group; Original magnification, ×200 (left). Graphical representation of the significant inhibitory effect of β-catenin siRNA transfection on cell migration and invasion (right). (**C**) The number of tumorspheres formed by cells transfected with β-catenin siRNA was significantly less than by the control siRNA or wild type cell group. (**D**) The number of colonies formed by cells transfected with β-catenin siRNA was significantly less than by the control siRNA or wild type cell group. Data represents experiments performed in triplicates and expressed as mean ± SD. *p < 0.05, **p < 0.01, ***p < 0.001.

To elucidate the significance of the niclosamide - associated dysregulation of β-catenin signaling axis in anticancer therapy, we examined the effect of transiently downregulating β-catenin activity on the expression and/or activity of the downstream effector genes, using the β-catenin -specific short interfering RNA (siRNA). Results of our western blot analysis showed that downregulation of β-catenin in tumorspheres derived from ALDH+ SCC4 cells induced remarkable downregulation of the expression levels of Cyclin D1, c-Myc and Snail proteins, as well as concurrent suppression of stemness factors, Oct4, Sox2 and Nanog protein expression level (Fig. 5A). This inhibitory effect of downregulating β-catenin on the selected proteins expression profile in the ALDH+ SCC4 tumorspheres was associated with the significant suppression of the invasion and migration potential in the β-catenin siRNA - transfected cells, compared with their control siRNA - transfected or wild type (WT) counterparts (p < 0.01 for β-catenin siRNA *vs* control siRNA or WT; Fig. 5B). Similarly, our tumorsphere formation assay showed significantly less number of tumorspheres formed by the β-catenin siRNA - transfected cells, compared with those formed by the control siRNA - transfected or wild type (WT) OSCC cells (p < 0.01 for β-catenin siRNA *vs* control siRNA or WT; Fig. 5C). Similar results were obtained for the colony formation capacity of the cells (Fig. 5D). This data suggests an association between the observed concurrent dowregulation of β-catenin, reduced expression of β-catenin downstream target genes and stemness markers, suppressed migration, invasion and colony formation, as well as loss of tumorsphere formation potential in the ALDH+ SCC cells.

### Niclosamide inhibits cisplatin-induced oral cancer stem cell enrichment, increases apoptosis, limits the colony-forming potential and enhances the sensitivity of the ALDH+ OSCC cells to cisplatin

**Figure 6:**
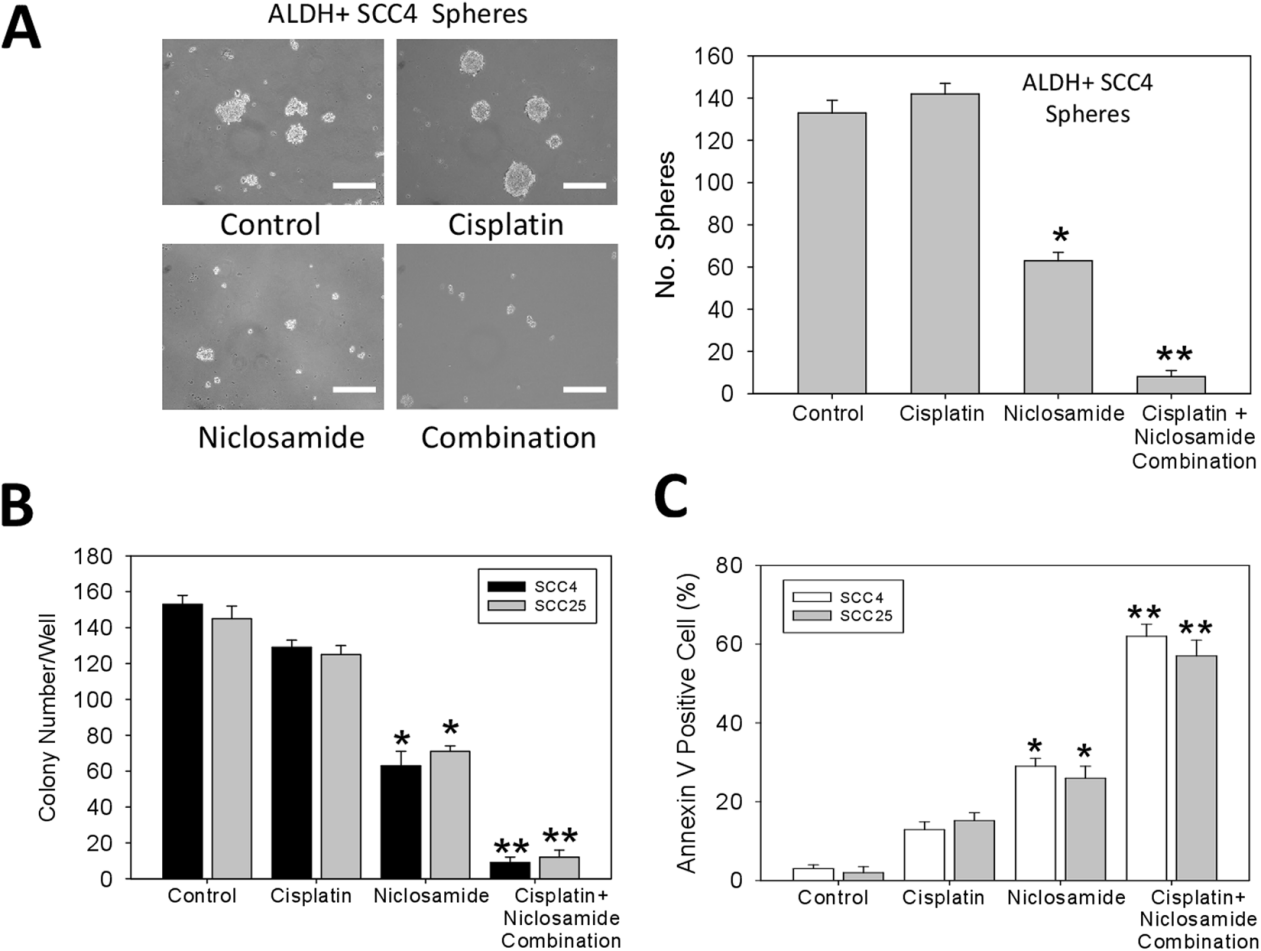
Niclosamide inhibits cisplatin-induced oral cancer stem cell enrichment, increases apoptosis, limits colony formation and enhances the sensitivity of the ALDH+ OSCC cells to cisplatin. (A) Photo images (*left*) and graphical quantitative data (*right*) of the tumorsphere-forming ability of the ALDH+ SCC4 cells after treatment with 10 μM cisplatin, 10 μM niclosamide or combination of 10 μM cisplatin and 10 μM niclosamide, compared with untreated control cells. (**B**) Graphical quantitative data of the number of colonies formed by the ALDH+ SCC4 or SCC25 cells after treatment with 10 μM cisplatin, 10 μM niclosamide or combination of 10 μM cisplatin and 10 μM niclosamide, compared with untreated control cells. (**C**) Graph showing the apoptotic effect of cisplatin alone, niclosamide alone, or cisplatin-niclosamide combination treatment in ALDH+ SCC4 or SCC25 cells. Data represents experiments performed in triplicates and expressed as mean ± SD. *p < 0.05, **p < 0.01, ***p < 0.001.

Since cisplatin is one of the preferred systemic chemotherapy for OSCC patients, we examined the effect of niclosamide on the activity of this standard agent for chemotherapy. After treatment of ALDH+ SCC4 cells with cisplatin in the presence or absence of niclosamide, our data indicate that for the cells treated with 10 μM of cisplatin alone generated more and larger tumorspheres compared to the untreated control group; conversely the 10 μM niclosamide-treated cells exhibited significantly suppressed ability to form tumorspheres (p < 0.05 *vs* control or cisplatin alone group), and the group treated with a combination of 10 μM cisplatin and 10 μM niclosamide significantly lost their ability to form tumorspheres (p < 0.01 *vs* control or cisplatin alone group) (Fig. 6A). In other experiments, while mild reduction in the colony forming ability of the 10 μM cisplatin- treated SCC4 and SCC25 cells were observed, 10 μM niclosamide treatment induced a significant inhibition of colony formation in the SCC4 (2.58-fold, p < 0.05) and SCC25 (2.06-fold, p < 0.05) cells, and cisplatin-niclosamide combination treatment induced a 17.22 - (p < 0.01) and 7.89 - (p < 0.01) fold reduction in the number of colonies formed by the SCC4 and SCC25 cell, respectively (Fig. 6B). In addition, to establish if the observed suppression of migration, invasion and colony formation, as well as the anti-CSC activity of niclosamide was associated with apoptosis in the OSCC cells, we analyzed niclosamide-induced cell death by Annexin-V apoptosis assay. The SCC4 and SCC25 cells were treated for 24 h with 10 μM of niclosamide or/and 10 μM of cisplatin, then Annexin-V labeling analysis was performed. We showed that while cisplatin treatment induced a mild (SCC4-8%; SCC25-15%) increase in the Annexin V positive cell population relative to the untreated control group, treatment with 10 μM of niclosamide induced 21% (p < 0.05) and 19% (p < 0.05) increase in apoptosis in SCC4 and SCC25 cells, respectively, when compared to the control group. Interestingly, when combined with 10 μM of cisplatin, 10 μM of niclosamide increased the number of Annexin V positive cells by 60% and 56% in the SCC4 and SCC25 cells, respectively (Fig. 6C); suggesting that niclosamide potentiates the anticancer activity of cisplatin.

## Discussion

Though considerable progress has been made in it diagnosis and treatment, OSCC is remains characterized by high incidence, low sensitivity to therapy, high risk of developing resistance to chemo- or radio-therapy, treatment failure and a relatively low 5-year survival rate^1–3^, making OSCC a medical challenge and necessitating the discovery or development of an effective treatment strategy. Until recently, most anticancer therapy assumed homogeneity and equal sensitivity of tumor cells to treatment agents or modality, thus, effectively targeting and inhibiting the growth and proliferation of bulk non-CSCs, while the small sub-population of OSCC cells referred to in this study as oral CSCs, escape the therapeutic effect of the chosen treatment regimen, resulting in cancer cell re-population, recurrence, enhanced aggressive phenotype, and increased likelihood of metastatic disease^5–7^. Thus, highlighting the need for therapeutic agents and strategies that effectively target and eliminate these CSCs in OSCC.

The relationship between cancer and embryonic tissues/cells has attracted a lot of attention after the development of hESCs and CSCs. Interestingly, most of the markers used to identify CSCs are derived from surface markers present on human embryonic stem cells (hESCs) or adult stem cells. Global analysis of gene expression networks further suggests that core pluripotency genes, such as NANOG, OCT4, SOX2, and MYC, are primary gene sets shared by both ESCs and CSCs^18^. Cancer stemness of tumor cells is measured *in vitro* by their ability to form tumorspheres and *in vivo* by their tumor-initiation capability in immune-compromised mice. Tumorspheres formation, tumor initiation, as well as the early steps of the metastatic process that require survival of the disseminating cells^19^. Aldehyde dehydrogenases (ALDHs) are important for maintenance and differentiation of stem cells as well as normal development. There is expanding evidence that ALDH expression increases in response to therapy and promotes chemoresistance and cancer stemness mechanisms in CSCs.

Exploiting our knowledge of the critical role of Wnt/β-catenin signaling in oral CSC activities^8,9^ and the suggested new role for the old antihelminthic niclosamide as an effective inhibitor of the Wnt/β-catenin signaling^10–14^, in the present study, we provide evidence that ALDH+ human oral squamous cell carcinoma cells are characterized by upregulated expression of pluripotency transcription factors and exhibit enhanced cancer stemness (Fig. 1). These findings are corroborated by documented evidence that ALDH1+ head and neck squamous cell carcinoma (HNSCC) cell lines and primary tissue samples exhibit enhanced tumor growth, migration and invasion capabilities, tumorsphere formation, resistance to chemotherapy and self-renewal ability^20^, especially as the ALDH1 represents a more specific marker of CSCs in HNSCC with an estimated 50.6–74.4% of ALDH1+ HNSCC cells express the cell-surface glycoprotein CD44 while only about 9.8–23.6% of CD44+ HNSCC cells exhibit high ALDH activity^21,22^, and this high ALDH activity correlates with upregulation of Sox2, Oct3/4 and Nanog in a population of spheroid-derived HNSCC cells^23^. Studies over the past decades have focused on the role of ALDH as a recognized CSCs marker and as a regulator of CSCs properties. Cojoc *et al*.^24^ analyzed the ALDH1A1 promoter with two core β-catenin/T-cell factor (TCF)-binding sites 5′-A/TA/TGGAAG-3′ through the chromatin immunoprecipitation assay and found that the β-catenin/TCF transcriptional complex is bound to this promoter. In general, they reported that the Wnt pathway, a significant pathway in CSCs, directly regulates ALDH1A1 level through β-catenin/TCF-dependent transcription as well as ALDH1A1 and β-catenin co-expression in prostate cancer cells. Zhao *et al*.^25^ evaluated breast cancer tissues from patients and found that tumour cells with high ALDH1 activity have low ALDH1A1 acetylation and are capable of self-renewal. They demonstrated that ALDH1A1 activity is inhibited by the acetylation of Lys 353 (K353) and that acetyl transferase P300/CBP-associated factor and deacetylase sirtuin 2 (SIRT2) are the enzymes responsible for the acetylation and deacetylation of ALDH1A1K353^25^. This finding clarifies the molecular mechanism involved in regulating ALDHs in CSCs from the perspective of transcriptional and post-translational regulation.

For the first time, to the best of our knowledge, we demonstrated that niclosamide potently suppresses the CSCs-like trait, including the migration, invasion, EMT, and colony formation potentials of ALDH -rich OSCC cell lines (Figs 2 and 3). This is consistent with the demonstrated ability of niclosamide alone or in combination with cisplatin to effectively reverse EMT and inhibit the CSC -like phenotype of wild type and cisplatin-resistant triple negative breast cancer (TNBC) MDA-MB-231 cell line, by disrupting the aberrant activation of Akt, ERK, and Src signaling pathway^26^. However, in this present study, we demonstrated that unlike in the TNBC cells, the anticancer activity of niclosamide against ALDH+ OSCC cells is mediated by the dysregulation of the Wnt/β-Catenin signaling pathway (Fig. 4), and similar to what is observed under downregulated β-Catenin conditions (Fig. 5). This is consistent with the demonstrated niclosamide-enhanced Fz1 endocytosis, downregulation of DVL2 protein, and inhibition of Wnt3A-stimulated β-catenin stabilization and LEF/TCF reporter activity in cells derived from mouse subcutaneous connective tissue^13^, targeting the Wnt co-receptor LRP6 on the cell surface of HEK293 human embryonic kidney cells^12^, as well as the confirmation of significant inhibition of Wnt/β-catenin signaling by TOPflash assay, decreased ALDH1A1 and LRP6 expression, increased cell death and reduced cell proliferation in human ovarian cancer cells treated with niclosamide^27^.

MMP-2 and MMP-9, which are secreted by invasive cancer cells, play important roles in cancer cell invasion and metastasis because tumor cells must cross the type IV collagen-rich basement of vessel walls to spread to other sites during oral cancer metastasis^28^. Nishio K. *et al*.^29^ revealed that MMP-2 activity is highly correlated with the risk for a relapse in oral squamous cell carcinoma patients. The transcription of MMPs and u-PA genes is regulated by upstream sequences, including motifs corresponding to NF-kB, activated protein-1, stimulatory protein-1 and polyoma virus enhancer activator-binding sites^30^. Therefore, it has been suggested that the suppression of NF-kB, c-Jun and c-Fos activities may inhibit tumor initiation, promotion and metastasis, and block the factors that bind to these regulatory elements, thus representing an appropriate approach to inhibit the synthesis of MMPs or u-PA. Here, we showed that niclosamide notably down-regulated the activities of MMP-2 and MMP-9, while the levels of TIMP-2 were enhanced (Fig. 3). To the best of our knowledge, this is the first scientific report relating the inhibitory effect of niclosamide on oral cancer invasiveness to decreased production of tumor metastasis-related proteins such as MMP-2 and u-PA.

Finally, we also demonstrated that niclosamide not only inhibit cisplatin-induced oral CSC enrichment, increase apoptosis, and limit the colony-forming potential, but it also significantly enhances the sensitivity of the ALDH+ OSCC cells to cisplatin (Fig. 6). Our data showing that the cells treated with cisplatin alone generated more and larger tumorspheres compared to the untreated control group is corroborated by the findings of Wang and his team, showing that cisplatin enriches the CSCs in non-small cell lung cancer (NSCLC) and that TRIB1/HDAC activity enhances the multidrug resistance of this lung CSCs^31^. Thus, our demonstrated ability of niclosamide alone or in combination with cisplatin to suppress this cisplatin-induced oral CSC enrichment is clinically relevant and may constitute another step towards establishing a more efficient anti-OSCC therapy in the clinic, especially as oral CSCs are implicated in pathogenesis, progression, metastasis, recurrence and poor prognosis.

**Figure 7:**
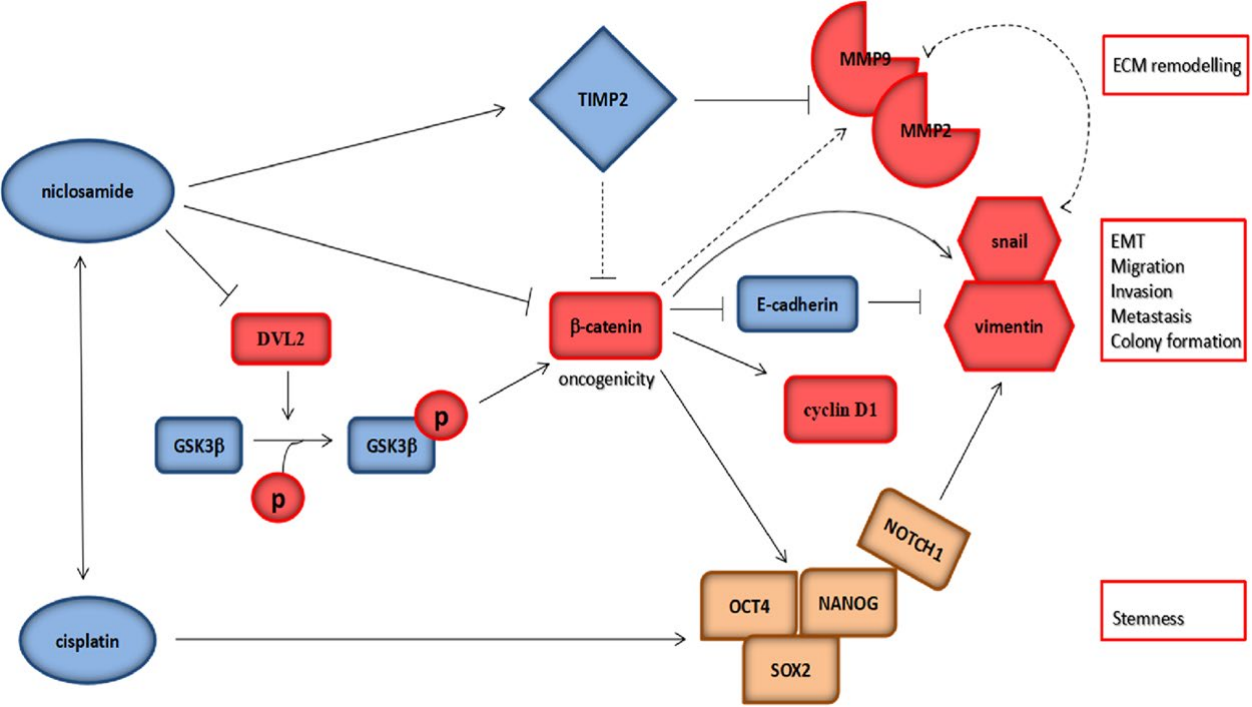
Pictorial Abstract. A schematic summary showing how nuclosamide through the dysregulation of the Wnt/β-catenin signaling pathway, inhibits the stemness of OSCC cells via the downregulation of pluripotency transcription factors, suppress oral CSC -associated EMT and metastasis by upregulating vimentin and snail, while downregulating E-cadherin, as well as limit EMT-related extracellular matrix (ECM) remodeling by modulation of TIMP2, MMP2 and MMP9. Similarly, nuclosamide suppresses the cisplatin-induced oral CSC enrichment and enhances the sensitivity of the ALDH+ OSCC cells to cisplatin.

In conclusion, as we depicted in our schematic summary (Fig. 7), we have provided evidence that the antihelminthic niclosamide inhibits cancer stemness, extracellular matrix remodeling, and metastasis through dysregulation of the Wnt/β-catenin signaling in OSCC. In essence, we showed that niclosamide through the dysregulation of the Wnt/β-catenin signaling pathway, inhibits the stemness of OSCC cells via the downregulation of pluripotency transcription factors, suppress oral CSC -associated EMT and metastasis by upregulating vimentin and snail, while downregulating E-cadherin, as well as limit EMT-related ECM remodeling by modulation of TIMP2, MMP2 and MMP9. Similarly, niclosamide suppresses the cisplatin-induced oral CSCs enrichment and enhances the sensitivity of the ALDH+ OSCC cells to cisplatin.

## Electronic supplementary material

Supplementary Information
