# Supplementary material for: The Antihelminthic Niclosamide Inhibits Cancer Stemness, Extracellular Matrix Remodeling, and Metastasis through Dysregulation of the Nuclear β-catenin/c-Myc axis in OSCC
Source: Sci Rep. 2018 Aug 24;8:12776. doi: 10.1038/s41598-018-30692-3 (PMC6109047; doi:10.1038/s41598-018-30692-3)
Supplement: Supplementary file 1 — Supplementary Information [file 41598_2018_30692_MOESM1_ESM.pdf]

SUPPLEMENTARY INFORMATION

The Antihelminthic Niclosamide Inhibits Cancer Stemness, Extracellular Matrix Remodeling, and Metastasis through Dysregulation of the Nuclear β-catenin/c-Myc axis in OSCC

Lin-Hong Wang1,#, Mei Xu2,#, Luo-Qin Fu3,4, Xiao-Yi Chen3,4, Fan Yang1*

1 Department of Stomatology, Zhejiang Provincial People's Hospital, People's Hospital of Hangzhou Medical College, Hangzhou 310014, Zhejiang Province, China

2 Special Department, Hangzhou Dental Hospital, Hangzhou 310013, Zhejiang Province, China

3 Clinical Research Institute, Zhejiang Provincial People's Hospital, People's Hospital of Hangzhou Medical College, Hangzhou 310014, Zhejiang Province, China

4 Key Laboratory of Tumor Molecular Diagnosis and Individualized Medicine of Zhejiang Province, Hangzhou 310014, Zhejiang Province, China

Corresponding author:

Dr. Fan Yang,

Department of Stomatology, Zhejiang Provincial People's Hospital, People's Hospital of Hangzhou Medical College, No. 158 Shangtang Road, Hangzhou 310014, China; Tel: +86-571-85893206, Fax: +86-571-85893206, E-mail: yangfan@hmc.edu.cn

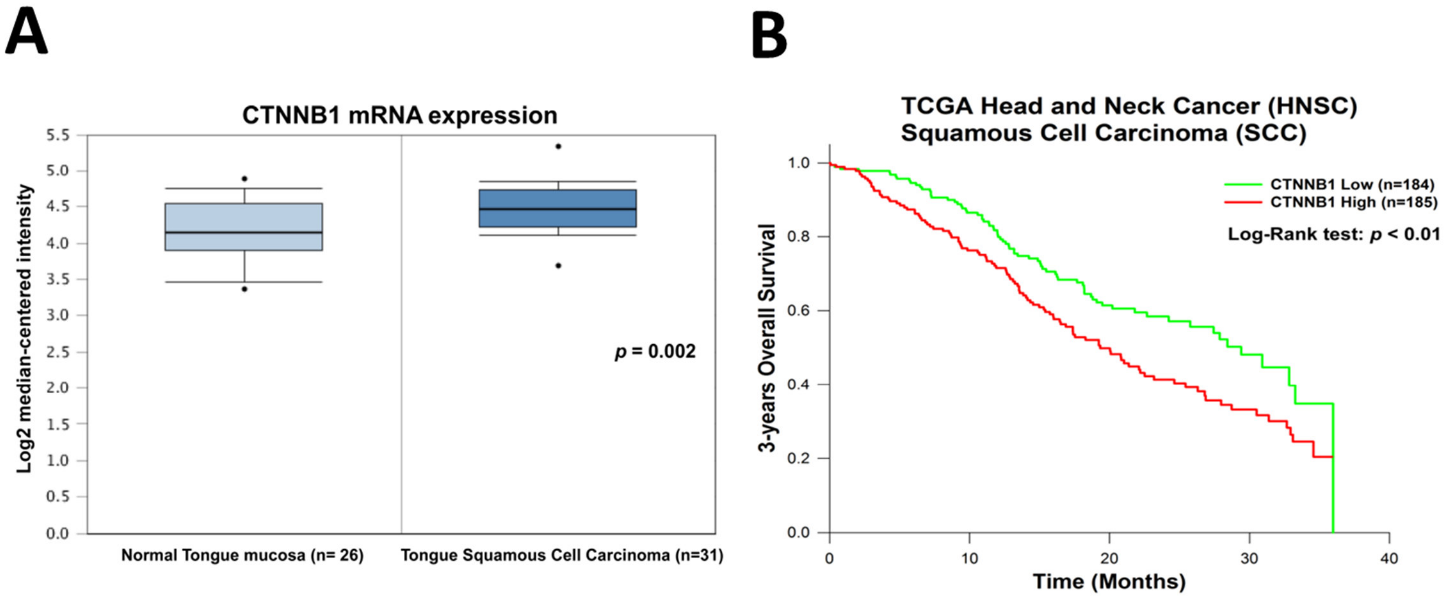

Supplementary Figure S1. Analysis of WNT/CTNNB1 mRNA expression in TSCC and HNSC using Oncomine and TGCA database. (A) Box-plot diagrams were analyzed to compare the WNT/CTNNB1 mRNA levels in normal tongue mucosa tissue with that in tongue squamous cell carcinoma using the Oncomine dataset. (B) Survival curves for HNSC in relation to WNT/CTNNB1 expression. Kaplan–Meier curves for TGCA database, stratified by high vs. low WNT/CTNNB1 expression.

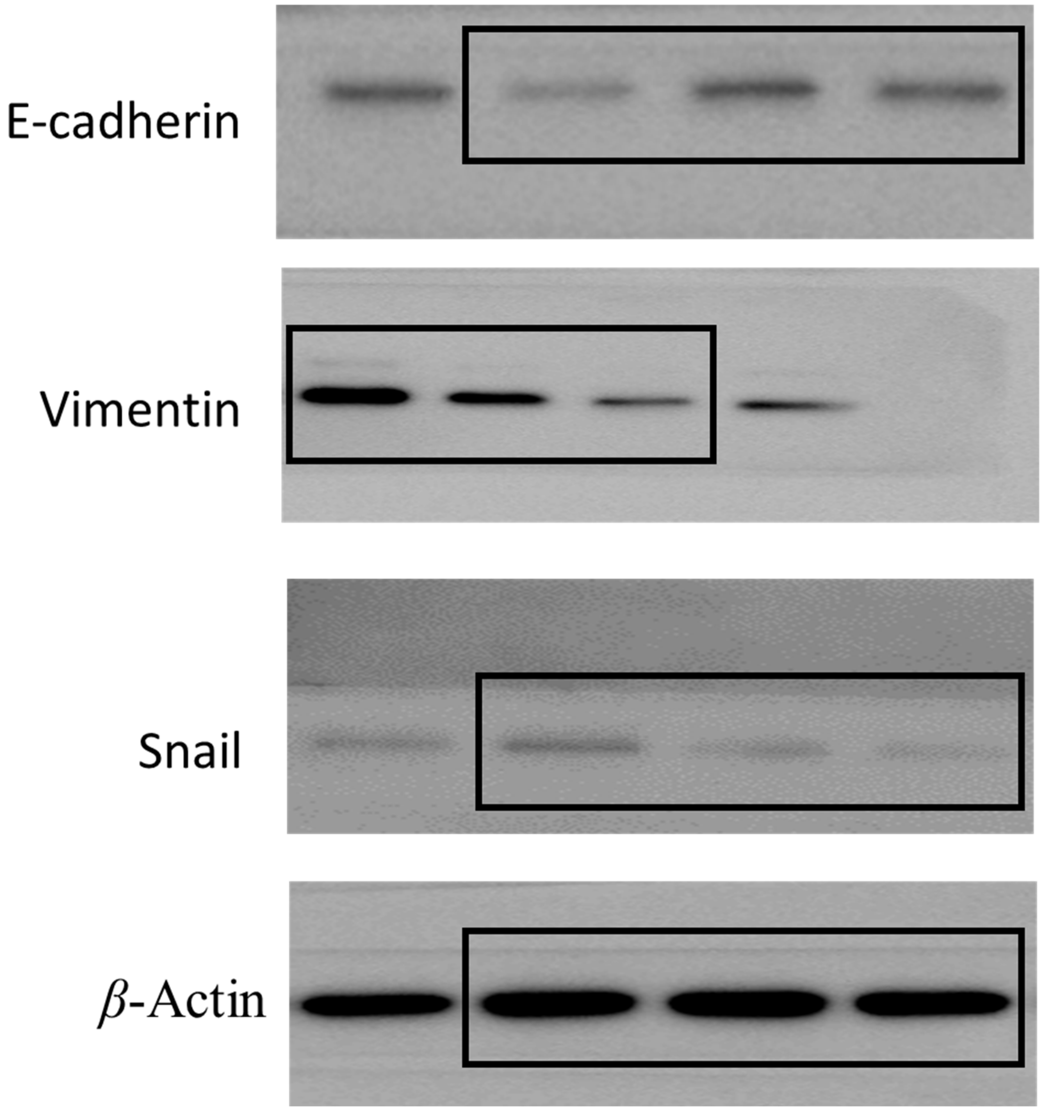

Supplementary Figure S2. Full-size blots of Figure 3D

MMP 2

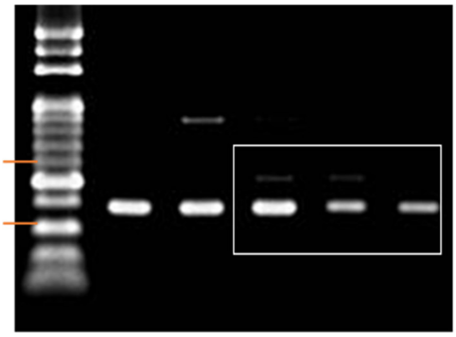

Annealing : 56 ˚c 30 sec
Elongation : 72 ˚c 30 sec
Cycle : 40

MMP 9

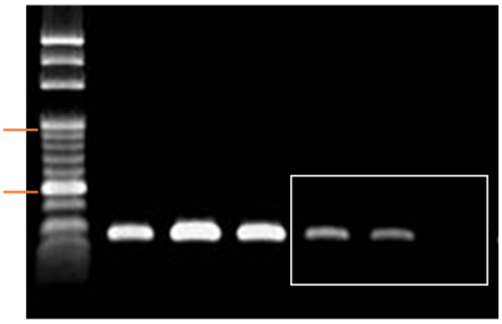

Annealing : 56 ˚c 30 sec
Elongation : 72 ˚c 30 sec
Cycle : 40

TIMP2

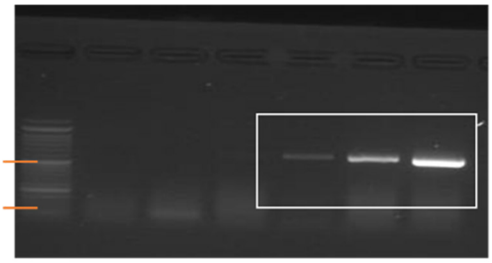

Annealing : 62 ˚c 30 sec
Elongation : 72 ˚c 30 sec
Cycle : 40

GAPDH

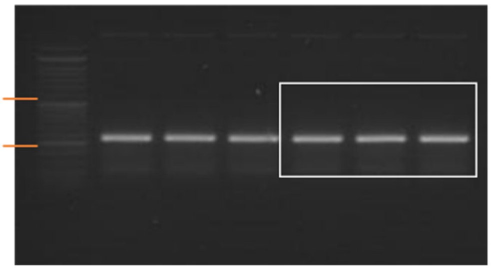

Annealing : 55 ˚c 30 sec
Elongation : 72 ˚c 30 sec
Cycle : 26

Supplementary Figure S3. Full-size blots of Figure 3E

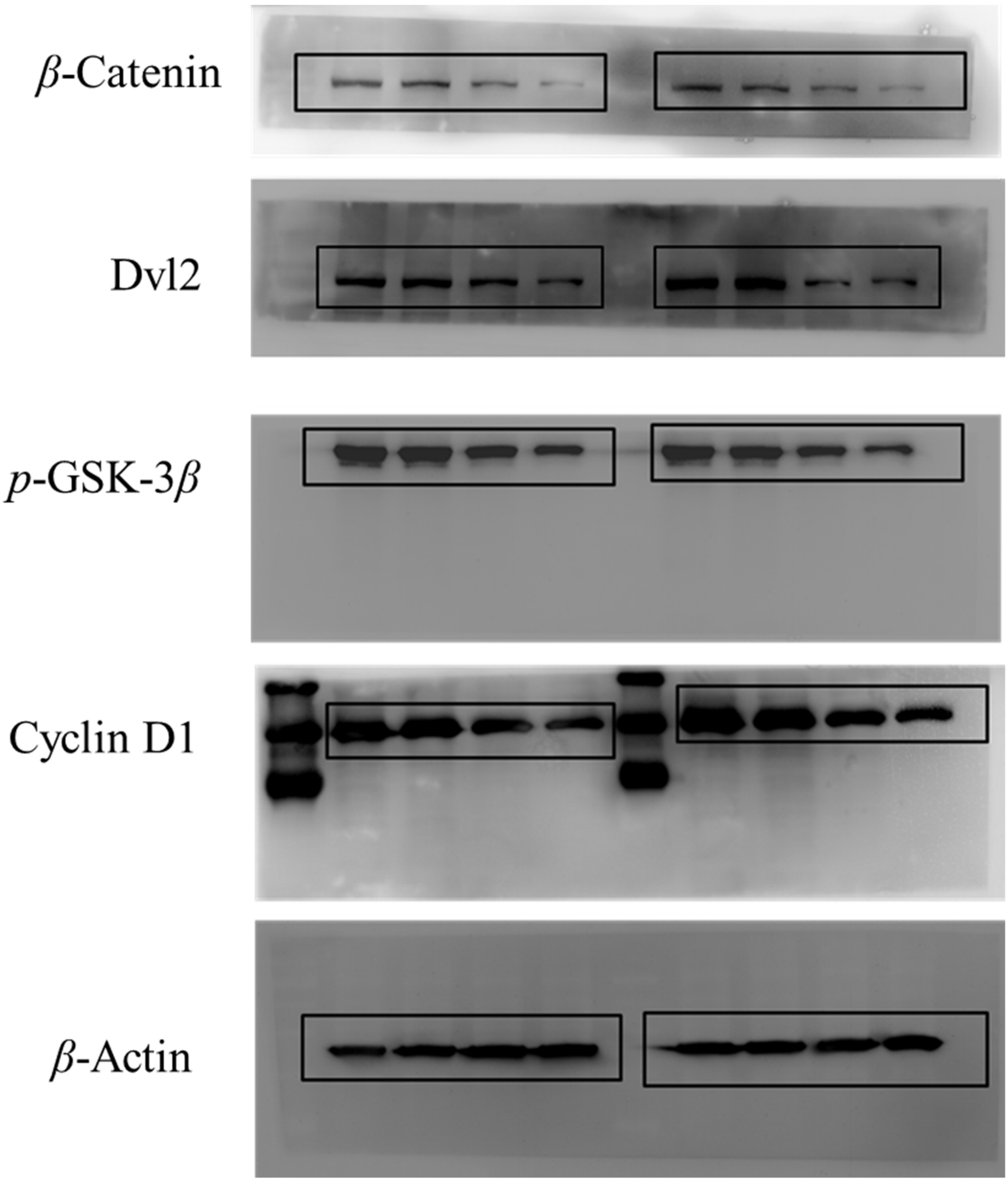

Supplementary Figure S4. Full-size blots of Figure 4C and 4D

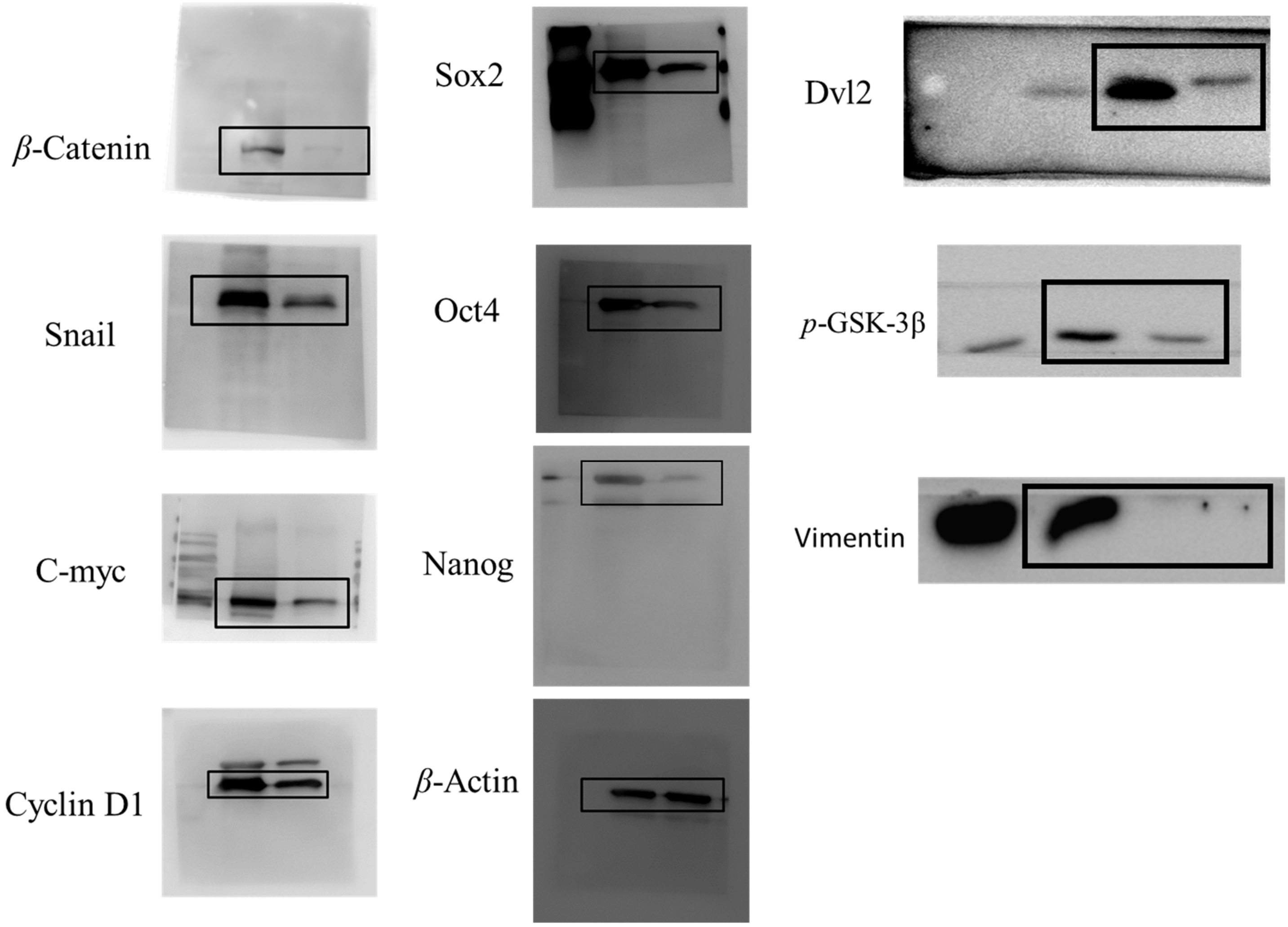

Supplementary Figure S5. Full-size blots of Figure 5A
